# The Lipodystrophy Syndrome in HIV-Infected Children under Antiretroviral Therapy: A First Report from the Central Africa

**DOI:** 10.1155/2019/7013758

**Published:** 2019-03-03

**Authors:** Honoré Kalombayi Tshamala, Loukia Aketi, Pierre Manianga Tshibassu, Mathilde Bothale Ekila, Eric Musalu Mafuta, Patrick Kalambayi Kayembe, Michel Ntetani Aloni, Joseph Diayisu Shiku

**Affiliations:** ^1^Division of Cardiology and Infectious Diseases, Department of Pediatrics, University Hospital of Kinshasa, Faculty of Medicine, University of Kinshasa, Kinshasa, Democratic Republic of the Congo; ^2^Division of Neurology, Nutrition and Gastroenterology, Department of Paediatrics, University Hospital of Kinshasa, School of Medicine, University of Kinshasa, Kinshasa, Democratic Republic of the Congo; ^3^Division of Infectious Diseases, Department of Internal Medicine, University Hospital of Kinshasa, Faculty of Medicine, University of Kinshasa, Kinshasa, Democratic Republic of the Congo; ^4^Division of Biostatistics and Epidemiology, School of Public Health, University of Kinshasa, Kinshasa, Democratic Republic of the Congo; ^5^Division of Hemato-Oncology and Nephrology, Department of Pediatrics, University Hospital of Kinshasa, Faculty of Medicine, University of Kinshasa, Kinshasa, Democratic Republic of the Congo

## Abstract

**Background:**

Despite the high prevalence of the HIV/AIDS, few studies focused on the prevalence of lipodystrophy in pediatric HIV patients on antiretroviral therapy (ARV) in sub-Saharan African countries. The aim of this study was to assess the prevalence and to identify the risk factors of metabolic disorders related to ARV therapy in this population.

**Methods:**

A cross-sectional study was completed in Kinshasa, the Democratic Republic of Congo. HIV-infected children aged between six and 18 years on ARV were consecutively recruited. For each case, two control children (one non-HIV infected child and one HIV-infected antiretroviral therapy-naïve child) were also recruited.

**Results:**

80 HIV-infected on ARV therapy children (group 1), 80 noninfected children (group 2) and 65 HIV-infected antiretroviral therapy-naïve children (group 3) were recruited. The frequency of lipoatrophy was not statistically different between group 1 (16.3%) and group 3 (21.5%). A significantly higher proportion of lipohypertrophy, hypercholesterolemia, and lactic acidosis was noted in children of group 1, compared to the controls (p<0.05). Mixed form was rarely observed in this series. The frequency of hypertriglyceridemia was not different between the 3 groups (p>0.05).

**Conclusion:**

Lipohypertrophy, hypercholesterolemia, and lactic acidosis emerge as a frequent metabolic disorders due to ARV therapy.

## 1. Introduction

The introduction of antiretroviral (ARV) therapy has greatly reduced morbidity and mortality and improved the quality and life expectancy of pediatric HIV patients [1]. However, lipodystrophy and dyslipidemia are common in pediatric HIV patients receiving ARV therapy and lipid profile may include an isolated elevation of triglycerides or cholesterol or a combination of both with various modifications of the concentration of cholesterol of low (LDL-c) or high (HDL-c) [2] density. These different risk factors may act as metabolic syndrome on the cardiovascular system [2]. However, studies conducted mainly in developed countries have reported conflicting results regarding the association between ARV therapy and the incidence of lipodystrophy and dyslipidemia in pediatric HIV patients [3]. These complications are particularly associated with the use of the protease inhibitors [2].

Sub-Saharan Africa contributes significantly to the high global rate of morbidity and mortality reported in HIV infection [4]. In the Democratic Republic of Congo (DRC), the overall prevalence of HIV was 4% and 37,000–52,000 pediatric HIV patients were less than fifteen years of age [4].

Despite this high prevalence of the disease and the risk of cardiovascular disease, very few studies focused on the prevalence of lipodystrophy in pediatric HIV patients on ARV therapy that have been reported in sub-Saharan African countries [5–7].

Further information on metabolic complications for HIV-infected children in this part of the world is urgently needed. We therefore conducted a cross-sectional study in pediatric HIV patients on ARV therapy. The aim of this study was to assess the prevalence of metabolic abnormalities and their phenotypic expression as lipodystrophy in HIV-infected children receiving ARVs compared with HIV-infected children not on ARVs and to HIV-negative children.

## 2. Methods

### 2.1. Study Setting and Design

The cross-sectional study was completed between March 2011 and September 2013 in five health institutions located in Kinshasa, the large city and capital of the DRC. The primary hospital was the University Hospital of Kinshasa and four secondary-care hospitals, Centre Médical Monkole, Centre de Santé AMOCONGO, Bomoyi Center of Kingasani, and Saint Joseph Hospital. These hospitals provide most of the nonprivate pediatric beds in the city.

HIV-infected children aged between six and 18 years on antiretroviral therapy for at least one year were consecutively recruited. For each case, two control children (one non-HIV infected child and one HIV-infected antiretroviral therapy-naïve child) matched for age, sex, and place of residence were also recruited into the study. A complete physical examination was carried out on each child by a pediatrician. Children were excluded where they had drugs which can induce hypertension or change carbohydrate metabolism.

### 2.2. Data and Sample Collection

The following formula was used to estimate the minimum size of the study population: n=Z^2^pq/d^2^. n = sample size; Z = confidence level at 95% (1.96); p = proportion of the target population with lipodystrophy. The prevalence of 18% found recently in the literature was the reference value for this study [8]. q = proportion of the target population without the characteristic of the study population (0.82); d = degree of accuracy (0.10). The minimum sample size was estimated at 56 children. In this study, our sample consists of 225 children who were recruited. Among them, there were 80 HIV-infected on ARV therapy children (group 1), 80 noninfected children (group 2), and 65 HIV-infected antiretroviral therapy-naïve children (group 3). Overall median age was 11.1 ± 3.4 years.

### 2.3. Clinical Features and Laboratory Analysis

The following information was recorded and analyzed: (i) demographic characteristics such as age and gender, (ii) triceps skinfold and Body Mass Index (BMI), (iii) clinical features, and (iv) laboratories investigations including total cholesterol, triglyceride, HDL, and LDL-cholesterol.

Height (cm) and weight (kg) were measured for each selected patient. BMI was calculated (BMI = weight/height2) (Kg/m2).

Diagnosis of HIV infection was made by using two antibody detection tests (Determine^R^ and Unigold^R^) for those older than 18 months, according to World Health Organization strategy (WHO) and the National Program against HIV (PNLS) of the DRC. The CD4 rate was measured in all HIV-positive children (HIV-positive cases and control group). HIV infection clinical stage was detected using the World Health Organization (WHO) clinical classification of HIV/AIDS in infants and children [9].

Total cholesterol, triglyceride, High Density Lipoprotein (HDL), and Low Density Lipoprotein- (LDL-) cholesterol were measured after 12 hours' fasting, and participants were rested for 30 minutes before a sample was taken and conserved on ice. The sample has processed within 30 minutes of the blood draw.

### 2.4. Definition

Dyslipidemia was defined as disorders of lipoprotein metabolism with level of the total cholesterol (TC), LDL-C, TG, and level of HDL-C.

Lipoatrophy, a loss of subcutaneous fat, was defined by the presence of sunken cheeks with prominent zygomatic arch or thin extremities with prominent veins with or without buttock atrophy. Lipohypertrophy was defined as an increased abdominal girth or fat accumulation “buffalo hump.” Mixed form was defined if there was one or more signs of sign for each of lipoatrophy and lipohypertrophy [10].

According to the National Cholesterol Education Program, hypercholesterolemia was defined as a cholesterol concentration > 200 mg/dl and hypertriglyceridemia as a cholesterol concentration > 150 mg/dl, high HDL as a serum HDL concentration > 85 mg/dL, and LDL as a serum LDL concentration ≥ 130 mg/dl [11].

### 2.5. Ethical Consideration

Since all participants were minors, they provided assent and their legal guardians provided consent for study participation. This consent procedure was reviewed and approved by the National Ethical Committee of the Public Health School of the University of Kinshasa, Kinshasa, DRC.

### 2.6. Data Management and Statistical Analysis

The validated data were entered into the computer using EPI Info version 6.0 (CDC, Atlanta, Georgia, USA). The analysis was performed using SPSS version 20.0. Continuous variables were expressed as mean ± standard deviation (SD) or medians (range) and categorical variables as relative frequency in percent. Associations between variables of dyslipidemia and HIV category were evaluated using chi-square tests, Student's t-tests, and Fisher's exact tests. The level of significance was set as p < 0.05.

## 3. Results

Characteristics of the study participants are presented in Table 1.

The majority of the children (66.7%) were between six and 12 years of age.

The majority of the children on ARV therapy (92.5%) were on the first-line regimen containing a nonnucleoside reverse transcriptase inhibitor (AZT + 3TC + NVP (or EFV)).

Lipoatrophy has been diagnosed in 16.3% in children on ARV therapy and 21.5% in HIV-infected antiretroviral therapy-naïve children. However, there was no statistically significant difference between the two groups.

A significantly higher proportion (15%) of subjects with lipohypertrophy were children on ARV therapy, compared to 7.7% in HIV-infected antiretroviral therapy-naïve children (Table 1).

Mixed form was rarely observed (Table 2).

A significantly higher proportion (7.5%) of subjects with hypercholesterolemia were children on ARV therapy. No case of hypercholesterolemia was reported in HIV-infected antiretroviral therapy-naïve children (Table 1).

Hypertriglyceridemia was present in 16.2% of children on ARV therapy and in 7% of HIV-infected antiretroviral therapy-naïve children. However, this difference was not significant (Table 3).

## 4. Discussion

The assessment of dyslipidemia is imperative because the national policy on ARV treatment has not yet assessed the metabolic side effects of these drugs in pediatrics. Probably this impairment is underreported in African children; poverty and the paucity of pediatricians and cardiologists in our midst should contribute to this fact.

In our cohort, the majority of the children were on the first-line regimen containing a nonnucleoside reverse transcriptase inhibitor (AZT + 3TC + NVP (or EFV)). Until 2010, the World Health Organization's (WHO) first-line regimen options for HIV-infected children included both stavudine and zidovudine. Although WHO guidelines no longer recommend it, many children in sub-Saharan Africa continue to receive stavudine as part of their cART regimen [12]. Furthermore, the difficulty of access to care explains the large number of children who do not receive antiretroviral treatment.

In this study, lipoatrophy was found in 16.3% in children on ARV therapy and 21.5% in HIV-infected antiretroviral therapy-naïve children. However, there was no statistically significant difference between the two groups. Children on ARV therapy face an increased risk of lipoatrophy due to this therapy particularly in regimen with nucleoside reverse transcriptase inhibitors leading to a loss of subcutaneous fat [13, 14]. The frequency of lipoatrophy in children on ARV therapy (16.3%) found in this study is similar to that reported in Tanzania with 19% of affected children [3, 5].

On the other hand, lipodystrophy has also been described in patients naive to HIV protease inhibitors [15].

A significantly higher proportion (15%) of subjects with lipohypertrophy were children on ARV therapy, compared to 7.7% in HIV-infected antiretroviral therapy-naïve children and 3.8% in noninfected children. These differences in the relative frequency of the lipohypertrophy prevalence between the three groups presumably arise from differences in the risk of exposure to ARV therapy, which influence the risk of development of lipohypertrophy. Our findings reported in this study were similar to those described elsewhere [5, 16].

Mixed form was rarely observed in our cohort and was similar to those reported by previous studies [10].

A significantly higher proportion of subjects with hypercholesterolemia were children on ARV therapy, in this cohort. The prevalence of hypercholesterolemia is high among HIV-infected children on ARV therapy, in this cohort. This finding is similar to previous reports from elsewhere [6, 17–19]. A recent study showed the association between the APOC3 genotype and hypercholesterolemia in an HIV-1-infected pediatric cohort exposed to ARV therapy [20].

The proportion of hypertriglyceridemia tended to be higher among children on ARV therapy than in the other two groups. In this study, the proportion of HIV-infected children with ARV therapy diagnosed with hypertriglyceridemia was 16%, which is similar to other worldwide studies that have reported 12% to 25% [16, 19, 21] but lower than the 29% to 83% reported in other pediatric cohorts [6, 22–26].

In a recent study, the authors found a protective effect of lipoprotein lipase polymorphisms against hypertriglyceridemia in children under ARV therapy [27].

## 5. Conclusion

Metabolic disorders encountered in HIV-infected children have been described. The emergence of lipohypertrophy, hypercholesterolemia, and lactic acidosis is more common in HIV-infected children on ARV therapy than in normal controls. Furthermore, it appears that one in 5 HIV-infected children had lipoatrophy. Population-based surveillance is required for accurate data that should be used to measure the magnitude, the evolution, and the pattern of metabolic disorders HIV-infected children living in the DRC. These clinical and biological markers may guide the clinician in the decision to initiate specific antiretroviral therapy in high resource settings.

## Figures and Tables

**Table 1:** Characteristics of the study population.

	HIV-INFECTED+ ART n=80	Naive HIV-INFECTED n=65	Negative HIV n=80

**Gender n, (**%**)**			
Boys	39 (48.6)	33 (50.8)	39 (48.6)
Girls	41 (51.4)	32 (49.2)	41 (51.4)
**Clinical stages n,(**%**)**			
Stage A	0 (0)	62 (95.4)	0 (0)
Stage B	66 (82.5)	3 (4.6)	0 (0)
Stage C	14 (17.5)	0 (0)	0 (0)
Stage D	0 (0)	0 (0)	0 (0)
**BMI (kg/m** ^**2**^ **)** *∗ * **n, (**%**)**			
Wasting < 15	23 (28.8)	22 (33.8)	10 (12.5)
Thinness (15-18.4	34 (42.5)	34 (52.3)	27 (33.8)
Normal ( 18.5-24.9)	22 (27.5)	9 (13.8)	42 (52.5)
Overweight (25-29.9)	0 (0)	0 (0)	0 (0)
Obesity ≥30	1 (1.2)	1 (1.5)	1 (1.2)
**Triceps skinfold** *∗∗ * **n,(**%**)**			
P< 50	66 (82.5)	57 (87.7)	30 (37.5)
P 50-90	13 (16.3)	8 (12.3)	49 (61.3)
P>90	1 (1.2)	0 (0)	1 (1.2)

^*∗*^p=0.001; ^*∗∗*^p>0.05.

**Table 2:** Distribution of lipodystrophy according to the type, location, and serostatus.

**Type**	**HIV+ ARVs** **n=80**	**Negative HIV** **n= 80**	**Naive HIV+** **n=65**	**p**

**Lipoatrophy**	**n=13(16.3)**	**n=0 (0)**	**n=14(21.5)**	0.000

(i) Ball of Bichat	2 (2.5)	0(0.0)	0 (0.0)	0.124
(ii) Fabric Fonte				
(a) Face	4 (5.0)	0(0.0)	6 (9.2)	0.007
(b) Members	4 (5.0)	0(0.0)	5 (7.7)	0.016
(c) buttocks	3 (3.8)	0(0.0)	3 (4.6)	0.085

**Lipohypertrophy**	**n=12 (5.0)**	**n=3(3.8)**	**n=5 (7.7)**	0.041

(i) neck	2 (2.5)	1 (1.2)	2 (3.0)	0.531
(ii) Buffalo humps	2 (2.5)	0 (0.0)	0 (0.0)	0.124
(iii) Breast adiposity	2 (2.5)	1 (1.2)	1 (1.5)	0.824
(iv) Abdominal obesity	6 (7.5)	1 (1.2)	2 (3.0)	0.093

**Mixed forms**	3 (3.8)	0 (0.0)	2 (3.0)	0.105

**Table 3:** Laboratory features in the study population according to serologic status and treatment.

	**HIV-Infected + ARVs n(**%**)**	**Negative HIV n(**%**)**	**Naive HIV-infected n(**%**)**	**p**
**Cholesterol (mg/dl)**				0.05
Hyper (> 200)	6 (7.5)	1 (1.2)	0 (0.0)	
**HDL-Cholesterol (mg/dl)**				0.25
Hyper (>150)	2 (2.5)	1 (1.2)	1 (1.5)	
**Triglycerides (mg/dl)**				0.136
Hyper (> 150)	13 (16.2)	4 (5.0)	7 (10.8)	
**Dyslipidemia**				0.52
Yes	74 (92.5)	77 (96.2)	60 (92.3)	
No	6 (7.5)	3 (3.8)	5 (7.7)	

## Data Availability

The data is available at the Department of Pediatrics of University of Kinshasa at the following contact: michelaloni2003@yahoo.fr.

## Additional Points

*Keynotes*. (i) Very few studies focused on the prevalence of lipodystrophy in paediatric HIV patients on ARV therapy that have been reported in sub-Saharan African countries. (ii) A significantly higher proportion of lipohypertrophy, hypercholesterolemia, and lactic acidosis was noted among HIV-infected children on ARV therapy, compared to the controls. (iii) Given the high rate of the HIV/AIDS in sub-Saharan Africa, there is a real need to prevent and manage metabolic disorders in HIV-infected children in our midst.
